# Multicomponent, nonpharmacological delirium interventions for older inpatients: A scoping review

**DOI:** 10.1007/s00391-019-01627-y

**Published:** 2019-10-18

**Authors:** Claudia Eckstein, Heinrich Burkhardt

**Affiliations:** 1 Network Aging Research, University of Heidelberg, Bergheimer Straße 20, 69115 Heidelberg, Germany; 2 Department of Geriatric Medicine, University Medicine Mannheim, Mannheim, Germany

**Keywords:** Hospitalization, Literature review, Cognitive decline, Epidemiology, Prevalence, Krankenhausaufenthalt, Literatur-Review, Kognitive Einschränkung, Epidemiologie, Prävalenz

## Abstract

**Background:**

Older people represent a risk group for acquiring or further development of delirium during hospitalization, therefore requiring suitable nonpharmacological delirium interventions.

**Objective:**

This scoping review analyzed nonpharmacological intervention programs for older inpatients with or without cognitive decline on regular or acute geriatric wards to present the range of interventions.

**Methods:**

A systematic literature search was conducted using scientific databases. A total of 4652 records were screened by two independent reviewers, leaving 81 eligible articles for full-text screening and 25 studies were finally included. Inclusion criteria were older patients ≥65 years in regular or acute geriatric wards and nonpharmacological multicomponent interventions.

**Results:**

More than a half of the included studies (14, 56%) recruited patients with pre-existing cognitive decline as part of the study population and 12% focused exclusively on patients with cognitive decline. On average 11 intervention components were integrated in the programs and two programs included full coverage of all 18 identified components.

**Conclusion:**

Only few programs were described for older inpatients and even fewer regarding pre-existing cognitive decline. The low numbers of interventions and data heterogeneity restricted the assessment of outcomes; however, delirium incidence, as reported by two thirds of the studies was reduced by nonpharmacological multicomponent interventions.

**Electronic supplementary material:**

The online version of this article (10.1007/s00391-019-01627-y) contains supplementary material, which is available to authorized users.

## Zusammenfassung

**Hintergrund:**

Ältere Menschen sind, insbesondere während eines Krankenhausaufenthaltes, gefährdet, Delirien zu entwickeln. Dies erfordert geeignete nonpharmakologische Delirinterventionen.

**Ziele:**

Dieser Scoping-Review analysierte nonpharmakologische Interventionsprogramme für ältere Patientinnen und Patienten mit und ohne kognitive Einschränkungen auf Normal- bzw. akut-geriatrischen Stationen und präsentiert die Reichweite und Vielfalt der integrierten Programmkomponenten.

**Methoden:**

Die systematische Literaturrecherche erfolgte mittels wissenschaftlicher Datenbanken. Zwei unabhängige Reviewer untersuchten 4652 Datensätze. Es wurden 81 Artikel in das Volltextscreening und 25 Studien endgültig in die Programmanalyse eingeschlossen. Die Einschlusskriterien waren Patientinnen und Patienten im Alter ≥65 Jahre auf Normal bzw. akut-geriatrischen Stationen und multikomponente, nonpharmakologische Interventionen.

**Ergebnisse:**

Es schlossen 14 Studien (56 %) Patientinnen und Patienten sowohl mit als auch ohne vorbestehende kognitive Einschränkungen in ihre Studienpopulation ein, während 12 % ausschließlich Erstere rekrutierten. Im Durchschnitt verfügen die Programme über 11 Interventionskomponenten. Zwei Programme integrierten alle von insgesamt 18 identifizierten Programmkomponenten.

**Fazit:**

Nur wenige Programme wurden für ältere stationäre Patientinnen und Patienten beschrieben, noch weniger für Personen mit bereits bestehenden kognitiven Einschränkungen.

Die geringe Anzahl von Interventionsprogrammen und die Datenheterogenität schränkte die Bewertung der Ergebnisse ein, jedoch wurde die Deliriuminzidenz, wie von zwei Dritteln der Studien berichtet, durch Mehrkomponenteninterventionen reduziert.

## Introduction

Delirium frequently occurs in older inpatients and constitutes a major and serious complication in acute care. It is associated with increased mortality [1–6] and worse outcomes, such as loss of personal independence [1, 2, 7, 8], significantly accelerated cognitive decline, a progressive pattern of dementia, increased hospital readmission and prolonged periods of hospitalization [5–12]. Old age [7, 8, 13–15] and predisposing (intrinsic) factors, such as chronic, especially cerebral diseases and geriatric syndromes, such as multimorbidity, frailty [16] and cognitive impairment represent high risks of developing delirium [2, 7, 11, 14, 16, 17]. Moreover, extrinsic factors, such as the clinical environment and conditions (e.g. surgery, infections, psychological stress, polymedication and disturbed day-night rhythm) may act as additional triggers [18, 19]. Consequently, a pronounced pre-existing vulnerability of individual patients who were predisposed by trigger factors in quantitative and qualitative terms, strongly correlates with delirium development as shown by the threshold model of Inouye et al. [19, 20]. Although the exact pathophysiological process remains unclear, the model indicates a multifactorial development of delirium, which requires multifactorial approaches for prevention and treatment. In the acute care setting, several delirium intervention programs provided by nonprofessionals, e.g. trained volunteers [20–27] or family members [28–30] are well-established and positively evaluated. Most of these programs focused on few selected intervention components to prevent delirium. In contrast, interventions to treat an already existing delirium are often disregarded. Consequently, these programs disclosed a limited range of interventions; however, professional expertise is required to ensure a more comprehensive delirium prevention and to also address delirium management and treatment. For this reason, this scoping focused on interventional programs provided by ward team professionals. In addition, these programs refer to the target group of older and endangered patients, but current evidence does not conclusively clarify whether this similarly applies to persons with cognitive decline, especially in the acute care setting [20, 21].

Previous reviews have also analyzed the effectiveness of nonpharmacological interventions. In the systematic overview by Abraha et al. [31] systematic reviews and meta-analyses and selected primary studies were included. Preventive effects were demonstrated in older inpatients ≥60 years, but not to treat delirium of older inpatients. The systematic review and meta-analyses by Martinez et al. [32], which exclusively included RTSs, also demonstrated significant results for older inpatients (e.g. reduced delirium incidence: relative risk, RR: 0.39, 95%, confidence interval, CI: 0.63–0.85, *p* = 0.001). The authors reported that the effects did not differ according to the prevalence of dementia. The extent to which the two populations with and without dementia differed within the included studies was not reported. Further two reviews, also focussing on acute care but not especially on older inpatients, demonstrated the effectiveness of nonpharmacological interventions. In the meta-analysis by Hshieh et al. [33] in which randomized and nonrandomized matched trials were included, significant effects were reported (e.g. delirium incidence: odds ratio, OR: 0.47, 95% CI: 0.38–0.58). The second review [34] (including quantitative studies) investigated critically ill inpatients and reported also an effectiveness of nonpharmacological interventions (e.g. a reduced delirium incidence: 24.7%, range 9.7–31.8%). Furthermore, from the empirical data of general delirium research it is known that the delirium syndrome can be reduced by 30–40% with multicomponent, nonpharmacological interventions [4, 20, 35–38]. This, of course, does not preclude the possibility of supplementary pharmacological interventions. In this context, nonpharmacological and pharmacological interventions often overlap and should be considered complementary. Noteworthy, an ongoing discussion addresses efficacy and safety of pharmacological approaches and some experts strongly discourage a pharmacological prevention on a routine base [19, 39–41]. Following the current findings from Siddiqi et al. [38], with the exception of atypical antipsychotic drugs (e.g. olanzapine), there is no clear evidence of the benefit of cholinesterase inhibitors, melatonin or antipsychotic drugs, e.g. haloperidol. The latter may be applied if there is psychometric overactivation or symptoms, such as hallucination. In this case, pharmacological treatment may supplement nonpharmacological treatment. In addition, drugs can also trigger a delirium syndrome, e.g. those with an anticholinergic potential [18], and sedatives and hypnotics have also shown potentially deliriogenic effects [42]. Remarkably, the multicomponent, nonpharmacological interventions have proven to be most effective [38, 43]. This is why the aim of this scoping review was to identify these nonpharmacological interventions for older inpatients with and without cognitive decline, as the previous reviews mentioned above did not all include these populations. Furthermore, this scoping focused, in contrast to previous reviews, on interventions performed by ward team professionals. The selection of the methodology of a scoping review is explained to the broad scientific question that is to be answered. In addition to previous reviews, which contained only specific study designs, this review was not limited to any study design.

## Objective and rationale

This scoping was conducted in order to identify delirium intervention programs that have been implemented and scientifically evaluated through clinical studies (which is why both terms “programs” and “studies” are used). The aim of this scoping was the identification of programs that are a) suitable to prevent and to manage/treat delirium of older inpatients b) with and without cognitive impairment or dementia on c) regular or acute geriatric hospital wards, d) provided by ward team professionals. The range of all integrated intervention components is described and the program effects are reported in terms of delirium incidence, prevalence, duration, severity and mortality. Based on the PICO scheme [44] the following three research questions were answered:

1. Which multicomponent nonpharmacological delirium intervention programs are described for older inpatients with and without pre-existing dementia or cognitive impairment for the acute setting in regular or geriatric hospital wards?
2. Which individual intervention components were integrated into the programs?
3. Which patient outcomes and effects are described for the programs in terms of delirium incidence, prevalence, duration, severity and mortality?

## Methods

### Protocol and registration

The registration and publication of the study protocol was deliberately omitted. At present, there are no established systems for scoping reviews that would allow or even justify registration. This review is a preliminary work for the intervention study DanA, registration at the German register of clinical trials (DIMDI) (DRKS-ID: DRKS00015755, protocol available at: https://www.drks.de/drks_web/navigate.do?navigationId=trial.HTML&TRIAL_ID=DRKS00015755; World Health Organization, international clinical trials registry platform: http://apps.who.int/trialsearch/Trial2.aspx?TrialID=DRKS00015755).

### Methodological framework

According to the methodological framework of Arksey and O’Malley [45] and the recommendations of Schmucker et al. [46], the scoping review was conducted in the following steps: (1) identifying the research question, (2) identifying relevant studies, (3) study selection, (4) charting the data and (5) collating, summarizing and reporting the results, based on the PRISMA-ScR checklist [47]. The scoping review adopts a classical narrative approach, which does not include quality assessment due to a lack of or insufficiently tested assessment tools [45, 46, 48–53] and was not aimed at performing a meta-analysis due to the heterogeneity of the interventions. Owing to the fact that this literature review tackles multicomponent interventions and answers broadly based scientific questions, the following methodological consequences are appropriately derived. (1) Due to the broad research topic, a scoping was performed instead of a classic systematic review. (2) The included study designs were not limited to RCTs. All quantitative and qualitative study designs were included, which considered previously defined inclusion and exclusion criteria, which are described below. Literature reviews themselves were not included.

### Eligibility criteria

The inclusion criteria were as follows: a) patients ≥65 years on regular or acute care geriatric wards (or similar ward designations), b) older individuals who have been treated in acute care settings for older people, c) with and without cognitive impairment/dementia, d) intervention: nonpharmacological interventions designed for professionals providing interventions on the ward. All studies that did not meet the defined inclusion criteria were excluded, e.g. long-term and home care, specialized departments, intensive care units (ICU), pharmacological interventions or interventions that were not provided by the ward team. The PICO criteria comparison was not predefined because all study designs were eligible (e.g. studies without control groups) and to avoid limiting the results, no outcome criterion was predefined.

### Information sources

The databases Cinahl, Cochrane library, Medline (via PubMed), PsychInfo and Web of Science were systematically searched (without time limits until 11 March 2019; selected languages English and German). Two authors were contacted to obtain the full study descriptions, one of which provided them. A third author, contacted by e‑mail, provided a previously unpublished manuscript (accepted for publication). Furthermore, a snowballing approach with backward citation tracking was conducted and a free web search was performed with Google Scholar, without identifying additional records.

### Search

After developing a suitable search strategy (according to Cochrane recommendations [54]), three complex search strings were generated using keywords, synonyms and MeSH terms, based on the described predefined inclusion/exclusion criteria (according to the PICO scheme [44]). The full electronic search strategy is presented in the online supplement No. 1. In order to review which intervention components are represented in current medical guidelines, a free online web search was conducted (keywords: delirium, interventions, prevention, treatment, management, medical, delirium, guideline, recommendation, elder, older, geriatric patients, dementia, cognitive decline, cognitive impairment, acute care, hospital, setting, regular, geriatric, non-ICU, ward). It was not intended to fully map the guideline-based evidence or to describe the levels of evidence, but to provide a basis for discussion on the extent to which the components correspond to current guidelines.

### Selection of sources of evidence

A title/abstract screening of 4652 identified records of interest was performed by two independent reviewers (authors 1 and 2). Out of these records 81 articles were retrieved as full-text versions and analyzed for eligibility and 25 studies were finally included in the dataset. A consensus on heterogeneous study inclusion could be reached between the two reviewers.

### Data charting process and data items

The data charting process was conducted by one reviewer (author 1), during the phase of provisional study inclusion (*n* = 81). The second reviewer (author 2) verified the datasets for completeness and correctness. The data extraction included information about bibliographic data, countries, objectives, study design, power calculation, setting, patient sample and number of participants, inclusion/exclusion criteria, inclusion of people with cognitive decline, performed in geriatric ward (yes/no), details about the type of program (based on direct indications or carefully and indirectly derived from the program characteristics without additional request to the authors), included components, data collection and analysis, measured endpoints, limitation and further information.

### Analysis and synthesis of results

Firstly, the identified programs for older inpatients were analyzed to determine to what extent they were also successfully tested on people with cognitive decline. Secondly, a further analysis served to collect and group all the intervention components described in the programs. The aggregated data were then used to highlight the range of the components. Due to the chosen scoping format and the inclusion of heterogeneous study designs with respect to methods, interventions and measured endpoints, a structured content analysis and synthesis were performed, and a predominantly descriptive summary was drafted.

## Results

### Selection of sources of evidence

**Fig. 1:**
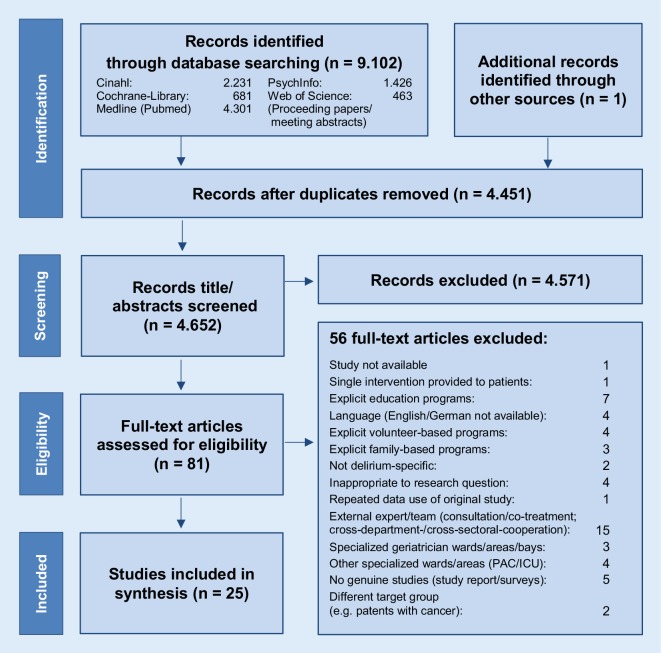
Presentation of the study selection

Based on the full-text screening, 25 studies were finally included. As shown in Fig. 1 [56] studies were excluded since the predefined inclusion criteria were not fully met: patients beyond regular wards (e.g. specialized, psychiatric, ICU wards), interventions, which were not provided by the ward team professionals (e.g. volunteers, cross-sectional services). All reasons for exclusion can be seen in the online supplement No. 2.

### Characteristics of sources of evidence

As shown in the online supplement No. 3 (“overview of included studies”), the selected studies were performed in 12 countries with 13 studies (52%) from Europe [55–67], eight (32%) from North America [68–75] and four (16%) from Australia [76–79]. Of the studies 11 (44%) were conducted using a pre-test and post-test design [55, 59, 60, 65, 68, 71–73, 75, 78, 79], six (24%) studies were performed as a prospective observational study [57, 62, 63, 67, 69, 70] and four (16%) studies were RCTs [56, 64, 66, 77]. Furthermore, one nonrandomized study (combined with pre-test and post-test design) [61], one propensity-matched cohort study [74], and two qualitative studies were conducted [58, 76]. Study participants ranged from *n* = 30 [76] to *n* = 19,949 [71] and all programs provided multicomponent, nonpharmacological interventions for older inpatients.

**Fig. 2:**
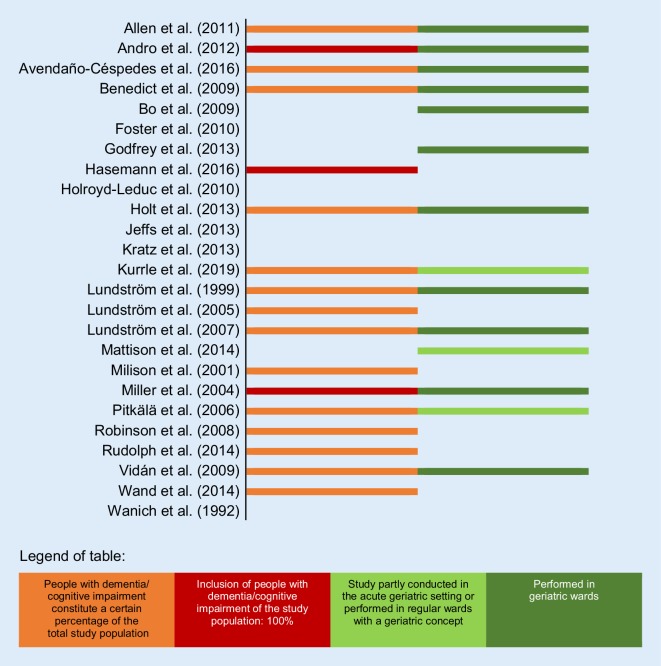
Delirium intervention programs for older inpatients

The age cohorts of older individuals varied considerably: one study recruited patients at the age of ≥60 years [69], eight (32%) included participants at the age of ≥65 years [56, 68, 70, 74, 76–79], two included patients at ≥69 years [66, 73], 7 (28%) recruited patients at ≥70 years [57, 59, 61, 63, 64, 67, 75], and one included patients at ≥75 years of age [55]. Of the included studies two used two age cohorts of older inpatients (80 years and older/70–79 years [71]; 64 years/74 years and older [72]). In addition, four (16%) studies without age limitation were included. The reason is on the one hand by the author’s descriptions of the setting as “elderly care” [58], “for elderly” [60] and “acute ortho-geriatric ward” [62] and on the other hand legitimized by the advanced mean age [65] (intervention group: 82 years, controls: 80 years). Of the studies 11 (44%) were performed in geriatric wards [55–58, 60, 62, 64, 67–69, 72], one study [79] was partially conducted in the geriatric setting (in 2 of 13 wards) and two studies (8%) held an intermediate position, as they were performed on regular units, but provided a geriatric care concept [66, 71]. Another 11 studies (44%) were conducted in nongeriatric units. In sum, only two programs were identified, which were tested exclusively on the subpopulation of older inpatients with cognitive decline in the geriatric setting ([55, 72]; Fig. 2).

### Syntheses of results

#### Programs tested on older inpatients with cognitive decline

Of the studies 14 (56%) also recruited patients with pre-existing dementia or cognitive impairment [56, 60, 62–69, 73, 74, 78, 79], whereas in other studies patients with severe cognitive decline were excluded. One study reported that significantly (*p* < 0.001) more patients with dementia (69.0%) developed delirium compared to patients without dementia (5.6%) [69]; however, in the investigation (*N* = 125) by Lundström et al. [63], no patient with dementia had delirium on day seven in the intervention ward, while in the control group four patients were delirious. Of the studies two examined especially the effect of the interventions on patients with dementia and found significant evidence for lower frequency [73] and shortened delirium duration in the dementia intervention group [64] compared to controls, three further studies (12%) were exclusively based on persons with dementia [55] or cognitive impairment [59] or both [72] (for further details see Table 1 and online supplement No. 4).

**Table 1:** Epidemiological patient outcomes in the 25 studies included in the review

Number of included studies →	1	2	3	4	5	6	7	8	9	10	11	12	13	14	15	16	17	18	19	20	21	22	23	24	25
**People with dementia/cognitive impairment constitute a certain percentage of the total study population**	X		X	X						X			X	X	X	X		X		X	X	X	X	X	
**Only people with dementia/cognitive impairment were included in the study population**		X						X											X						
*Incidence (measured in 17 out of 25 studies)*																									
Effects of reduced delirium incidence with statistical significance		X	X		X					X		X		X		X					X		X	X	
Effects of reduced delirium incidence demonstrated with tendency	X			X				X	X		X							X							X
No effects of reduced delirium incidence have been found																									
*Prevalence (measured in 6 out of 25 studies)*																									
Effects of reduced delirium prevalence with statistical significance			X										X												
Effects of reduced delirium prevalence demonstrated with tendency														X	X										
No effects of reduced prevalence have been found						X										X									
*Duration of delirium (measured in 9 out of 25 studies)*																									
Effects of reduced delirium duration with statistical significance										X					X	X		X							
Effects of reduced delirium duration demonstrated with tendency			X					X															X		
No effects of reduced delirium duration have been found											X			X											
*Severity (measured in 6 out of 25 studies)*																									
Effects of reduced severity of delirium with statistical significance			X							X								X							
Effects of reduced severity of delirium demonstrated with tendency																									
No effects of reduced severity of delirium have been found								X^a^			X												X		
*Mortality (measured in 13 out of 25 studies)*																									
Effects of reduced delirium-associated mortality with statistical significance															X										
Effects of reduced delirium-associated mortality demonstrated with tendency	X																								
No effects of reduced delirium-associated mortality have been found			X						X	X				X		X	X	X		X			X	X	X

Number of included studies: 1 Allen et al. (2011) [68], 2 Andro et al. (2012) [55], 3 Avendaño-Céspedes et al. (2016) [56], 4 Benedict et al. (2009) [69], 5 Bo et al. (2009) [57], 6 Foster et al. (2010) [76], 7 Godfrey et al. (2013) [58], 8 Hasemann et al. (2016) [59], 9 Holroyd-Leduc et al. (2010) [70], 10 Holt et al. (2013) [60], 11 Jeffs et al. (2013) [77], 12 Kratz et al. (2015) [61], 13 Kurrle et al. (2019) [79], 14 Lundström et al. (1999) [62], 15 Lundström et al. (2005) [63], 16 Lundström et al. (2007) [64], 17 Mattison et al. (2014) [71], 18 Milisen et al. (2001) [65], 19 Miller et al. (2004) [72], 20 Pitkälä et al. (2006) [66], 21 Robinson et al. (2008) [73], 22 Rudolph et al. (2014) [74], 23 Vidán et al. (2009) [67], 24 Wand et al. (2014) [78], 25 Wanich et al. (1992) [75]

*X* = criterion fulfilled, *no entry* = not published in this paper

^a^Study reported positive effects when one out of four wards had been excluded from the statistical calculation

#### Identified intervention components

**Fig. 3:**
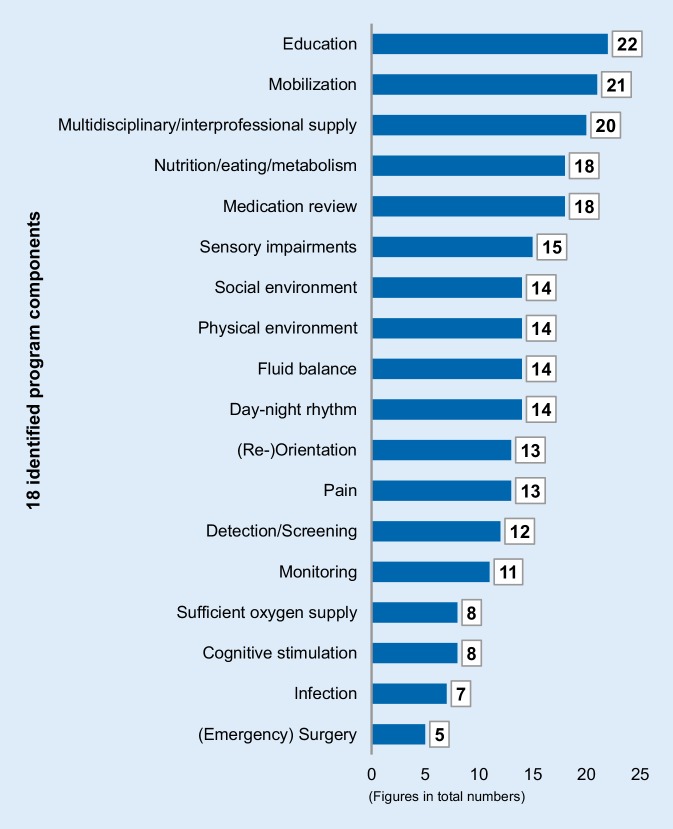
Frequency distribution of intervention components

A total of 18 nonpharmacological intervention components from the included studies were identified. Programs contained between 5 [65, 77] and 18 components [59, 79]. Staff-education was the most represented component (88%), followed by mobilization (84%). In addition to these 18 intervention components, illustrated in Fig. 3, several programs included other individual interventions, e.g. computerized order systems, caregiver booklets, standardized diagnostic procedures and further geriatric or risk assessments (see online supplement No. 4). Additional individual interventions varied between the programs. Supplementary pharmacological interventions are not comprehensively described in the studies. The extent to which these individual components are used by the programs for both prevention and treatment/management of delirium cannot be fully answered. Therefore, only a provisional allocation to the priority areas of prevention and/or management/treatment was made as mentioned above. Of the programs 11 (44%) were identified that focused primarily on delirium prevention [55, 57, 58, 60, 61, 67, 69, 70, 73, 74, 77], one of which was assigned to manage delirium [66], while 13 (52%) intervention programs covered both aspects of prevention and management without specifying interventions between hypoactive and hyperactive forms.

#### Intervention components represented in current medical guidelines

A total of 10 international guidelines [11, 80–88] were identified including recommendations for older patients on regular or geriatric wards based on a keyword-centered online search. The 18 identified program components for prevention and management of delirium are represented in the analyzed guidelines. In addition, the guidelines provide further recommendations for treatment (for details see online supplement No. 5). Overall, it can be stated that within the guidelines there is a considerably degree of agreement and an overall consistency in the following seven components: detection, fluid balance, infection, medication review, monitoring, and sensory impairment. Remarkably, only half of the programs addressed the component detection/screening for clinical routine and were otherwise used just for diagnosis during the study phase. Lowest accordance with a 100% guideline recommendation was noted for component monitoring*, *only mentioned in 44% of the programs. Regarding this aspect, observation of patients should be applied in routine clinical care, and a broader future discussion might address the extent of clinical observations actually capturing delirium-specific indicators. Although nine out of ten guidelines recommended pain and sufficient oxygen supply, these components are rather underrepresented in the programs (50% and 32%, respectively). This may be an indication that these areas, if not optimized, are still underappreciated as delirium trigger factors. Further six components are recommended by 80% of the analyzed guidelines: staff-education, (emergency) surgery, mobilization, mode of healthcare supply (five of those [11, 80, 83, 87, 88] endorsed explicitly multidisciplinary/interprofessional supply)*, *nutrition/eating/metabolism, and physical environment*. *The recommendations on education and mobilization are the most common program components and reveal their increasing importance in delirium care. In contrast, the component (emergency) surgery was embedded in only 20% of the programs, possibly due to the fact that just a few surgical departments were included. Depending on the primary medical problem or diagnosis, postoperative patients are also treated in regular or geriatric units, illustrating the additional importance of this component outside surgical fields. Particularly nonelective surgical procedures [85] are associated with an increased delirium risk. The components day-night rhythm and social environment were recommended in 70% of guidelines, also representing integral parts of intervention programs (>50%), and cognitive stimulation was considered in 50% of the guidelines vs. 32% in the programs.

#### Epidemiological patient outcomes and program effectiveness

One study focused exclusively on team endpoints and did not investigate patient outcomes [58]. All other studies focused on patient outcomes. Some of these additionally investigated team-related endpoints that are not discussed in this paper. Patient-related outcomes extend to both delirium-specific epidemiological variables, such as incidence, prevalence, duration of delirium, severity, and mortality and to corresponding delirium variables, such as complications, medication, ICU transfers, discharge destination, length of stay and other outcomes as described in detail in the online supplement No. 4. In most cases, the measured endpoints were objective and health economic-related parameters. Patient-related outcomes, e.g. aspects of well-being [72] or quality of life, however, were less investigated. Based on the underlying research questions, this review does not focus on the differentiated reporting of all patient outcomes but intends to give a comprehensive overview of important epidemiological variables. Incidence was the most frequently represented epidemiological outcome (17, 68%), followed by mortality (13, 52%), duration (9, 36%), severity, and prevalence of delirium (each 6, 24%).

##### Incidence and prevalence.

The endpoint incidence of delirium was investigated with heterogeneous results. In 10 (40%) programs, all intervention groups demonstrated a statistically significant reduced delirium incidence. Andro et al. [55] found a declining incidence in the intervention phase (15.5% vs. 5.3%) and also Avendaño Céspedes et al. [56] (41.4% vs. 14.3%, *p* = 0.039), and Bo et al. [57] (RR 0.90, *p* < 0.001). Holt et al. [60] reported a decline of delirium (from 13.3% to 4.6%) and Kratz et al. [61] stated higher initial incidence (20.2% vs. 4.9%). Lundström et al. [62] measured a reduced postoperative delirium incidence after program implementation (61.3%. vs. 30.6%), and also Lundström et al. [64] reported a lower incidence of delirium in the intervention group (75.3% vs. 54.9%, *p* = 0.003). Robinson et al. [73] reached a declined delirium incidence (*p* < 0.001), Vidán et al. [67] reported lower incidence of delirium (*p* = 0.005), and also Wand et al. [78]. A total of seven studies (28%) reported tendencies of a reduced delirium in the intervention groups but lacked statistical significance [59, 65, 68–70, 75, 77]. The six studies that measured prevalence of delirium presented results in various aspects. Avendaño Céspedes et al. [56] showed a decreased delirium prevalence in the intervention group (RR 0.54). Foster et al. [76] initially measured that 10 out of 30 patients were delirious. After program implementation, an identical delirium prevalence rate was measured. In contrast, Kurrle et al. [79] reported an increase in delirium coding from 27% to 79%. Lundström et al. 1999 [62] and 2005 [63] reported only a tendency towards delirium reduction in the intervention group in comparison to controls. In the study by Lundström et al. 2007 [64] no significant difference between intervention and controls was reported.

##### Duration of delirium.

The majority of the nine studies (36%) investigating duration reported a shortened delirium, four (16%) with statistical significance. Holt et al. [60] measured a reduced (*p* = 0.002) length of delirium during the first seven days in the intervention group (IG, *n* = 152) vs. control group (CG, *n* = 210) as also demonstrated by Lundström et al. 2005 [63] (*p* < 0.001) on day seven (IG: *n* = 63 vs. CG: *n* = 62). Lundström et al., 2007 [64] indicated a reduced delirium (*p* = 0.009) among postoperative patients (IG: *n* = 102 vs. CG: *n* = 97) and Milisen et al. [65] also reported a shortened delirium (IG: *n* = 60 vs. CG: *n* = 60) (*p* = 0.03). Further three studies (IG: *n* = 21 vs. CG: *n* = 29; IG: *n* = 138 vs. CG: *n* = 130; IG: *n* = 170 vs. CG: *n* = 372) also showed a shortened duration, but without statistical significance [56, 59, 67]. In contrast, one study (IG: *n* = 59 vs. CG: *n* = 214) based on historical study comparisons reported no effects [62] and another study (IG: *n* = 305 vs. CG: *n* = 343) noted a slightly prolonged duration in the intervention group [77].

##### Severity.

Regarding the severity of delirium, no homogeneous results were reported. In three out of six studies empirical evidence was given. Avendaño Céspedes et al. [56] found an overall reduced severity in the intervention group (*p* = 0.040), albeit including an increased mean severity per day. Holt et al. [60] reported a positive effect in the intervention group (*p* = 0.005) similar to Milisen et al. [65] (*p* = 0.0049). In three further studies, no effects regarding the severity of delirium were demonstrated [59, 67, 77].

##### Mortality.

Out of 13 studies two, each based on one measurement point, demonstrated a reduced mortality rate in the intervention cohort. Allen et al. [68] reported fewer in-hospital deaths in the intervention group and Lundström et al. [63] showed a significantly lower mortality (*p* = 0.03) during hospital stay. Further two studies [56, 65] stated a slightly decreased, nonsignificant mortality. Other authors [70] reported on a fatal case among those enrolled an four further studies [67, 71, 75, 78], based on one measurement point did not show an effect. In two other studies, no effects were measured first in hospital and after six months [60] or after one year [66]. Another study [64] with three time points (during hospitalization, four‑month and 12-month follow-ups) also found no effects at all. Using an approach of historical comparison, one study [62] reported very different findings. The authors stated an in-hospital mortality of the intervention cohort of 2.0% vs. the two control groups of 2.7% and 5.8%. In the six‑month follow-up, however, a higher mortality rate in the intervention group in comparison to controls (16.2% vs. 12.6%) was reported.

##### Program effectiveness in terms of epidemiological patient outcomes.

Based on the data of this scoping review, nonpharmacological interventions have hardly any effect on mortality. Statements regarding program effectiveness on prevalence, severity and duration of delirium are only possible to a very limited extent, since there are very heterogeneous findings. The incidence, measured in 17 out of 25 studies, is the most likely indicator of program effectiveness. In two studies that measured the endpoint incidence the programs were exclusively tested on people with cognitive decline, one of those studies [55] showed a significantly decreased and the other [59] a nonsignificantly decreased incidence. As shown in Table 1, 11 other studies, which measured the endpoint delirium incidence, included also patients with cognitive decline in the study population. In seven of those studies a statistically significant reduction in incidence was demonstrated, in three at least a statistically nonsignificant reduction, while in one study no effects could be demonstrated; however, it could not be conclusively stated, not least due to a lack of subgroup analyses, to what extent the group with cognitive decline actually benefits from the intervention programs.

## Discussion

### Summary of evidence

#### Programs for older inpatients

This scoping review emphasized that there are a number of relevant delirium intervention programs for older inpatients, which have been implemented and scientifically evaluated through clinical studies. Most programs targeted the criteria of older patients. Approximately half of the studies included participants with pre-existing cognitive decline but were restricted to cohort subgroup analyses. Only three exclusively focused on cognitive decline [55, 59, 72].

#### Intervention components

Of 25 analysed programs, only two [59, 79] covered all 18 identified intervention components. While an earlier review [34] covered both generalized (e.g. nutrition) and operationalized individual interventions (e.g. warm drinks), this scoping focused on the identification of generalized intervention components to be considered in regular or geriatric wards. The three other relevant reviews also followed a more general approach. In one of those reviews, a selective search strategy was pursued [33], focussing on a certain program type. This explains why this scoping is more in line with the research of Martinez et al. [28] and Abraha et al. [31] regarding the identified program components. Remarkably, in none of the four relevant previous reviews was monitoring (of endangered and delirious patients) described as an essential intervention component, which is also recommended in all 10 guidelines analyzed.

Several components that are recommended in medical guidelines have only partially been considered in the programs and most studies did not state in clear terms, whether interventions for hypoactive or hyperactive delirium forms had been applied. Based on the data of this scoping, no patterns can be derived from the number, selection and combination of intervention components that would allow conclusions to be drawn about their effectiveness. Another essential aspect should also be stressed, namely the manner in which intervention components are provided by healthcare teams.

#### Epidemiological patient outcomes and program effectiveness

The epidemiological endpoints defined by studies were heterogeneous, thus limiting statements on delirium incidence, prevalence, duration, severity and mortality. The evidence can most likely be derived from the measured endpoint incidence. As in previous reviews [32–34], a reduced delirium incidence could be demonstrated by nonpharmacological interventions. In summary, the basis of currently available data does not allow conclusive assessments of effects of nonpharmacological intervention components on delirium incidence and other epidemiological patient outcomes; however, it appears obvious that nonpharmacological multicomponent interventions may be beneficial for older inpatients, probably also those with pre-existing cognitive decline. Further research to investigate the effect on cognitively impaired patients would be needed to provide actual evidence.

### Strengths and limitations

An obvious limitation of previous reviews is their restriction regarding study designs, interventions and outcomes, so that here particular importance was assigned to these issues, thus trying to confer specific strength of reviewing. This scoping review covers a variety of nonpharmacological delirium intervention programs for older inpatients on regular or acute geriatric wards. In this respect, the envisioned review goals could be achieved, namely to describe the epidemiological impact of programs and to identify the integrated components.

It is possible that some programs could not be included due to predefined inclusion criteria. In addition, the comprehensive English-German search strategy covered relevant databases; however, it cannot be completely ruled out that further or unpublished studies or studies in other languages may exist that have not been identified.

A controversially discussed limitation of scoping reviews is seen in providing answers to broad scientific questions without any evaluation [45], thus raising the question about the necessity of more critical appraisals. In fact, no validated instruments are available for a critical review appraisal, so that authors claimed the future development of assessment tools [50, 52]. In contrast, for systematic reviews, based on a specific and narrowly focused scientific research question, critical appraisal is obligatory [54]. Notably, appraisals have also been generated for other types of evidence synthesis [90, 91], which were not an alternative to this scoping review, since they followed conceptual algorithms that serve to develop a theory or framework. This review, however, aimed to identify programs and interventions components for older inpatients and to provide orientation for the development of future delirium programs.

Another putative limitation may be given by the exactness of assigning the components cognitive stimulation, orientation, and social environment (i.e. similar measures were assigned to different components from the perspective of study authors). This issue was here settled by carrying out intervention assignments exactly on the basis of the author specifications (still comprising some uncertainty arising from heterogeneous statements). In addition, restrictions on the validity of epidemiological data may also be mentioned. The findings presented are based on heterogeneous assessments or diagnostic procedures, measured in different time periods and therefore only comparable to a limited extent.

### Conclusion and implications for future research

This review provides an overview of nonpharmacological delirium intervention programs for older inpatients with and without cognitive decline in regular or acute geriatric hospital settings. On the one hand, several programs and program components were found in order to improve delirium care. On the other hand, considerable gaps were detected regarding delirium care of older inpatients with cognitive decline, so that intervention programs should be further developed. Due to currently low numbers of subgroup analyses on dementia vs. nondementia cohorts, their benefit has so far remained unclear. Although the programs comprised heterogeneity, a large number of components from various fields were integrated, including the component monitoring. This indicates that delirium care needs an increasing awareness and the consideration of medical guidelines. Furthermore, it requires complex structures and professional expertise. The epidemiological data, although not conclusive, provide indications of the impact of the intervention. Moreover, due to their heterogeneous composition, the impact of the intervention components cannot be deduced from certain patterns. In this respect, this scoping confirms that nonpharmacological multicomponent interventions can also have an impact on older individuals without being able to describe the underlying mechanisms of action. For a description of the impact, not only the intervention component would need to be considered in future research, but also the way as well as the quality and quantity of its application.

## Practical conclusion

There are only a small number of delirium intervention programs explicitly described for older inpatients in acute care and even a lower number for those with cognitive decline. Delirium care is a complex task integrating a broad range of components from support of organ function (e.g. oxygen supply) to more complex tasks such as re-establishing a structured environment and daily activities. The analysis of components with respect to representation in programs and guidelines found a variety of patterns but also underlines the multifactorial approach within all programs; however, this multifactorial approach remains a challenge in daily care for older patients in hospitals. Improving delirium prevention and management depends not only on awareness for different intervention components in the multifactorial approach or completeness of their total range but also on strategies to implement and integrate those different facets. Care teams therefore should be encouraged to focus on aspects that help to strengthen multifactorial and multiprofessional team approaches, such as team conferences; however, the efficacy of such aspects remains to be evaluated and there is still a remarkable research gap in that respect.

## Caption Electronic Supplementary Material

1. Search strategies and search terms used in Medline (via PubMed)

2. Reasons for study exclusion

3. Overview of included studies

4. Identified multicomponent, nonpharmacological interventions and patient outcomes

5. Recommendations (overview) of relevant medical delirium guidelines

## Abbreviations

ICU: Intensive care units
PICO: Patient/problem‐intervention‐comparison‐outcome
PRISMA‐ScR statement: Preferred reporting items for systematic reviews and meta-analyses extension for scoping reviews
RCT: Randomized controlled trial
WHO: World Health Organization
