# Supplementary material for: Multicomponent, nonpharmacological delirium interventions for older inpatients: A scoping review
Source: Z Gerontol Geriatr. 2019 Oct 18;52(Suppl 4):229–42. doi: 10.1007/s00391-019-01627-y (PMC6820613; doi:10.1007/s00391-019-01627-y)
Supplement: Supplementary file 1 — 1. Search strategies and search terms used in Medline (via PubMed) [file 391_2019_1627_MOESM1_ESM.pdf]

ZGG_Zeitschrift für Gerontologie und Geriatrie
Multicomponent, non-pharmacological delirium interventions for older inpatients. A scoping review.

Supplement No. 1

Claudia Eckstein[1], Heinrich Burkhardt[2]
1 Network Ageing Research, University of Heidelberg, Germany.
2 Department of Geriatric Medicine, University Medicine Mannheim, Germany.

Seach strategies and search terms used in Medline (via PubMed) *

Delirium<br><br>+ Old age<br>+ Hospital<br>+ With intervention<br>- Without dementia/ cognitive impairment	Delirium<br><br>+ Old age<br>+ Hospital<br>- Without intervention<br>+ Without dementia/ cognitive impairment	Delirium<br><br>- Without old age<br>+ Hospital<br>+ With intervention<br>+ With dementia/ cognitive impairment
(<br>Delirium[Mesh] OR<br>Deliri*[tiab] OR<br>acute confusion*[tiab] OR<br>"metabolic encephalopathy"[tiab] OR<br>"clouding of consciousness"[tiab]<br>)<br>AND<br>(<br>("cognition disorders"[mh] OR<br>cognitive impair*[tiab] OR<br>cognitive dysfunction*[tiab] OR<br>cognitive disorder*[tiab] OR<br>neurocognitive disorder*[tiab] OR<br>cognitive decline[tiab])<br>OR<br>(dementia[mh] OR<br>dementia[tiab] OR<br>alzheimer*[tiab] OR<br>"Lewy Body"[tiab])<br>)<br>AND<br>(<br>aged[mh] OR<br>ageing[tiab] OR<br>elder*[tiab] OR<br>older[tiab] OR<br>old age*[tiab] OR<br>"old people"[tiab] OR<br>senior*[tiab]<br>)<br>AND<br>(<br>"Hospitals"[Mesh] OR<br>"Hospitalization"[Mesh] OR<br>ward*[tiab] OR<br>unit*[tiab] OR<br>clinic[tiab] OR<br>clinics[tiab] OR<br>clinical[tiab] OR<br>hospital*[tiab]<br>)<br>NOT<br>(<br>"Intensive Care Units"[Mesh] OR<br>"Residential Facilities"[Mesh] OR<br>"Hospices"[Mesh] OR<br>"Cancer Care Facilities"[Mesh] OR<br>"Hospitals, Maternity"[Mesh] OR<br>"Hospitals, Pediatric"[Mesh] OR<br>"Hospitals, Psychiatric"[Mesh]<br>)<br>AND<br>(german[la] OR english[la])<br>NOT<br>(animals[mh] NOT humans[mh])	(<br>Delirium[Mesh] OR<br>Deliri*[tiab] OR<br>acute confusion*[tiab] OR<br>"metabolic encephalopathy"[tiab] OR<br>"clouding of consciousness"[tiab]<br>)<br>AND<br>(<br>aged[mh] OR<br>ageing[tiab] OR<br>elder*[tiab] OR<br>older[tiab] OR<br>old age*[tiab] OR<br>"old people"[tiab] OR<br>senior*[tiab]<br>)<br>AND<br>(<br>treatment[tiab] OR<br>intervention[tiab] OR<br>strateg*[tiab] OR<br>concept*[tiab] OR<br>"Tertiary Prevention"[Mesh] OR<br>"Secondary Prevention"[Mesh] OR<br>"Primary Prevention"[Mesh] OR<br>prevent*[tiab] OR<br>"prevention and control" [sh] OR<br>manag*[tiab] OR<br>Path[tiab] OR<br>Paths[tiab] OR<br>pathway*[tiab] OR<br>Program*[tiab]<br>)<br>AND<br>(<br>"Hospitals"[Mesh] OR<br>"Hospitalization"[Mesh] OR<br>ward*[tiab] OR<br>unit*[tiab] OR<br>clinic[tiab] OR<br>clinics[tiab] OR<br>clinical[tiab] OR<br>hospital*[tiab]<br>)<br>NOT<br>(<br>"Intensive Care Units"[Mesh] OR<br>"Residential Facilities"[Mesh] OR<br>"Hospices"[Mesh] OR<br>"Cancer Care Facilities"[Mesh] OR<br>"Hospitals, Maternity"[Mesh] OR<br>"Hospitals, Pediatric"[Mesh] OR<br>"Hospitals, Psychiatric"[Mesh]<br>)<br>AND<br>(german[la] OR english[la])<br>NOT<br>(animals[mh] NOT humans[mh])	(<br>Delirium[Mesh] OR<br>Deliri*[tiab] OR<br>acute confusion*[tiab] OR<br>"metabolic encephalopathy"[tiab] OR<br>"clouding of consciousness"[tiab]<br>)<br>AND<br>(<br>("cognition disorders"[mh] OR<br>cognitive impair*[tiab] OR<br>cognitive dysfunction*[tiab] OR<br>cognitive disorder*[tiab] OR<br>neurocognitive disorder*[tiab] OR<br>cognitive decline[tiab])<br>OR<br>(dementia[mh] OR<br>dementia[tiab] OR<br>alzheimer*[tiab] OR<br>"Lewy Body"[tiab])<br>)<br>AND<br>(<br>treatment[tiab] OR<br>intervention[tiab] OR<br>strateg*[tiab] OR<br>concept*[tiab] OR<br>"Tertiary Prevention"[Mesh] OR<br>"Secondary Prevention"[Mesh] OR<br>"Primary Prevention"[Mesh] OR<br>prevent*[tiab] OR<br>"prevention and control" [sh] OR<br>manag*[tiab] OR<br>Path[tiab] OR<br>Paths[tiab] OR<br>pathway*[tiab] OR<br>Program*[tiab]<br>)<br>AND<br>(<br>"Hospitals"[Mesh] OR<br>"Hospitalization"[Mesh] OR<br>ward*[tiab] OR<br>unit*[tiab] OR<br>clinic[tiab] OR<br>clinics[tiab] OR<br>clinical[tiab] OR<br>hospital*[tiab]<br>)<br>NOT<br>(<br>"Intensive Care Units"[Mesh] OR<br>"Residential Facilities"[Mesh] OR<br>"Hospices"[Mesh] OR<br>"Cancer Care Facilities"[Mesh] OR<br>"Hospitals, Maternity"[Mesh] OR<br>"Hospitals, Pediatric"[Mesh] OR<br>"Hospitals, Psychiatric"[Mesh]<br>)<br>AND<br>(german[la] OR english[la])<br>NOT<br>(animals[mh] NOT humans[mh])

* The search strategies and search terms used in the other databases Cinahl, Cochrane-Library, PsychInfo and Web of Science can be requested from the authors (e-mail: eckstein@nar.uni-heidelberg.de, heinrich.burkhardt@umm.de).
