# Supplementary material for: Multicomponent, nonpharmacological delirium interventions for older inpatients: A scoping review
Source: Z Gerontol Geriatr. 2019 Oct 18;52(Suppl 4):229–42. doi: 10.1007/s00391-019-01627-y (PMC6820613; doi:10.1007/s00391-019-01627-y)
Supplement: Supplementary file 2 — 2. Reasons for study exclusion [file 391_2019_1627_MOESM2_ESM.pdf]

ZGG_Zeitschrift für Gerontologie und Geriatrie
Multicomponent, non-pharmacological delirium interventions for older inpatients. A scoping review.

Claudia Eckstein[1], Heinrich Burkhardt[2]
1 Network Ageing Research, University of Heidelberg, Germany.
2 Department of Geriatric Medicine, University Medicine Mannheim, Germany.

Supplement No. 2

Reasons for study exclusion

Exclusion category	Study		Quantity
**Study requested, but not available**	(1)	Fick et al._2015	1
**Single intervention**	(2)	Goldberg et al._2014	1
**Exclusively education programm/measurement**	(3)<br>(4)<br>(5)<br>(6)<br>(7)<br>(8)<br>(9)	Cooley et al._2016<br>Palmer et al._2014<br>Pizzacalla et al._2015<br>Tabet et al._2005<br>Van der Steeg et al._2014<br>Wilkerson et al._ 2014<br>Yevchak et al._2012	7
**Language**<br>(Englisch/German not available)	(10)<br>(11)<br>(12)<br>(13)	Bonaventura et al._2007 (Italian language)<br>Chi et al._2015 (Chinese language)<br>Hwang et al._2015 (Korean language)<br>Navarrete et al._2016 (Spanish language)	4
**Exclusively volunteer-based approaches**	(14)<br>(15)<br>(16)<br>(17)	Caplan et al._2007<br>Gorski et al._2017<br>Sandhaus et al._2010<br>Steunenberg et al._2016	4
**Exclusively family-based approaches**	(18)<br>(19)<br>(20)	Martinez et al._2012<br>Paulen et al._2016<br>Rosenbloom-Brunton et al._2010	3
**Not delirium-specific**	(21)<br>(22)	Rubin et al._2006<br>Stenvall et al._2012	2
**Inappropriate to research question**	(23)<br>(24)<br>(25)<br><br><br>(26)	Borgardus et al._2003 (long-term-effects)<br>Pretto et al._2009 (workload nurses)<br>Van Velthuijsen et al._2017<br>(Retrospective data collection of provided interventions, not based on a program)<br>Yevchak et al._2017 (qualitatively derived themes)	4
**Repeated data use of original study**	(27)	Pitkälä et al._2008	1
**External expert team**<br>Intervention approaches that were not privided by health care professionals of a ward team: consultation, liaison, co-treatment, cross-department cooperation, cross-sectoral cooperation provided by external experts (with or without the combination of voluntary support)	(28)<br>(29)<br>(30)<br>(31)<br>(32)<br>(33)<br>(34)<br>(35)<br>(36)<br>(37)<br>(38)<br>(39)<br>(40)<br>(41)<br>(42)	Angel et al._2016<br>Bakker et al._2014<br>Baldwin et al._2004<br>Björkelund et al._2010<br>Booth et al._2019<br>Cole et al._1994<br>Cole et al._2002<br>Deschodt et al._2012<br>Inouye et al._1999<br>Inouye et al._2000<br>Marcantonio et al._2001<br>Mouchoux et al._2011<br>Naughton et al._2005<br>Yoo et al._2014<br>Zaubler et al._2013	15
**Specialized geriatrician wards/areas** with unique architecture or surroundings, special fittings, e.g. "delirium room"	(43)<br>(44)<br>(45)	Chong et al._2014<br>Goldberg et al._2013<br>Mudge et al._2012	3
**Other specialized wards/areas**<br>(Day clinics, rehabilitation facilities, psychiatric hospital, long-term care, home care, oncology, palliative care, hospice, emergency, intensive care, maternity/child care)	(46)<br>(47)<br>(48)<br>(49)	Balas et al._2014<br>Bergmann et al._2005<br>Ferguson et al._2018 (mixed Setting)<br>Kolanowski et al._2016Ka	4
**No original studies**<br>(study report/surveys/summeries)	(50)<br>(51)<br>(52)<br>(53)<br>(54)	Hasemann et al._2010<br>Hasemann et al._2013<br>Menzies et al._2012<br>Inouye et al._1993<br>Inouye et al._2004	5
**Different target groups**<br>(e.g. people with cancer)	(55)<br>(56)	Chen et al._2011<br>Chen et al._2017	2
**Summary**			**56**
