# Supplementary material for: Multicomponent, nonpharmacological delirium interventions for older inpatients: A scoping review
Source: Z Gerontol Geriatr. 2019 Oct 18;52(Suppl 4):229–42. doi: 10.1007/s00391-019-01627-y (PMC6820613; doi:10.1007/s00391-019-01627-y)
Supplement: Supplementary file 3 — 3. Overview of included studies [file 391_2019_1627_MOESM3_ESM.pdf]

ZGG_Zeitschrift für Gerontologie und Geriatrie
Multicomponent, non-pharmacological delirium interventions for older inpatients. A scoping review.

Supplement No. 3

Claudia Eckstein[1], Heinrich Burkhardt[2]
1 Network Ageing Research, University of Heidelberg, Germany.
2 Department of Geriatric Medicine, University Medicine Mannheim, Germany.

Overview of included studies

No.	Study	Country	Objectives	Design	Setting[1]	Patient-sample[1]	Duration	In-/exclusion criteria	Inclusion of PwD[2]/ MCI[3] ?	Performed in geriatric ward?
1	Allen et al. (2011)	USA	Early detection/treatment/ prevention of delirium	Pre- and post-test design	Acute-care for elders	Geriatric population; age 65 and over; pre-phase: n=102; post-phase: n=97	Not stated	Not stated	(X)	X
2	Andro et al. (2012)	France	Evaluation of demented hospitalized patients	Pre- and post-test design	Acute-care geriatric	Geriatric population; age 75 and over; pre-phase: n=367; post-phase: n=372	07/2008-12/2009; 18 months	Inclusion: several forms of dementia	X	X
3	Avendaño-Céspedes et al. (2016)	Spain	Analysis of nurse-led interventions (patient outcomes: incidence/duration/severity of delirium, and feasibility)	RCT[4] (power-calculation conducted)	Acute-geriatric ward	Geriatric population; age 65 and over; IG[5]: n=321; CG[6]: n=29	10/2013-02/2014; 5 months	Exclusion: severe cognitive decline	(x)	X
4	Benedict et al. (2009)	USA	Investigation of the effectiveness of delirium prevention protocols, based on modified NEECHAM (Neelon et al. 1996) tool	Prospective observational study	Acute-care of elders´ unit	Population; age 60 and over; IG[5]: n=70 (mean age: 78,9 years); CG[6]: n=35 (mean age: 78,1 years)	01/2006-04-2006; 4 months	Exclusion: delirium, long-term care, drugs	(X)	X
5	Bo et al. (2009)	Italy	Investigation of acute-geriatric ward, compared with acute-general medical ward: which ward is associated with reduced delirium incidence	Prospective observational study	Acute-geriatric ward/acute-medical ward	Geriatric population; age 70 and over; IG[5]: n=121; CG[6]: n=131	01/2007-04/2007; 4 months	Exclusion: delirium, psychiatric and neurologic disorders, language, alcohol		X
6	Foster et al. (2010)	Australia	To implement a best practice approach to assess, to manage and to prevent delirium	Qualitative: action research	2 acute-medical wards	"High-risk" population; age 65 and over (history of fall, 3 medications or 1 opiate, memory loss or confusion, visual/hearing impairment, severe illness (n=30)	2008; 6 months	Exclusion: patients without risk profile		
7	Godfrey et al. (2013)	UK	Development of a novel delirium, focused in the implementation process	Qualitative:, participatory action research	3 hospitals, elderly care	Population: elderly, not specified	Not described in detail; 14 months			X
8	Hasemann et al. (2016)	Switzer-land	Determination of the effects of DemDel, a comprehensive delirium management program for older inpatients in acute-care with cognitive impairment	Pre- and post-test design (power-calculation conducted)	4 general medical wards	Population; age over 70 years and older; IG[5]: n=138; CG[6]: n=130	01/2009-08/2009; 01/2010-08/2010; 2 x 8 months	Exclusion: language, blind/deaf patients, terminal/neurological illness, coma, drugs, alcohol; inclusion: all patients with signs of cognitive impairment	X	
9	Holroyd-Leduc et al. (2010)	Canada	Implementation/evaluation of an evidence-based electronic care pathway, which incorporates multicomponent delirium strategies	Interrupted design (prospective cohort study)	2 orthopaedic units	Population: age over 65 years and older with hip fractures; IG[5]: n=170; CG[6]: n=173	10/2008-08/2009; 11 months	Exclusion: non-native speakers, fractures caused by motor vehicle crashes		
10	Holt et al. (2013)	UK	Examination of the effect of a multicomponent, delirium prevention intervention	Pre- and post-test design	3 care wards (specialized for elderly)	Population: "elderly" without limitation IG[5]: n=187 (mean age: 85,8 years, SD: ±5.39); CG[6]: n=249 (mean age: 85,01 years, SD: 6.03)	10/2007-03/2008; 6 months 08/2008-01/2009; 6 months	Exclusion: delirium; language;inclusion: admitted as emergency	(X)	X
11	Jeffs et al. (2013)	Australia	Comparison between an intervention program (with exercise, mobilisation, and orientation) and usual care to prevent delirium	RCT[4] (power-calculation conducted)	2 acute-medical wards	Population: age over 65 years or older; IG[5]: n=305; CG[6]: n=344	05/2005-12/2007; 32 months	Exclusion: severe dysphasia, communication impossible, death expected within 24 h, isolation, contraindication mobilisation, stroke/ICU		
12	Kratz et al. (2015)	Germany	Investigation of a prospective intervention with psycho-geriatric liaison* on surgical wards	Non-randomized, combined with pre- and post-test design	2 general surgical wards	Population: age over 70 years and older; prevalence phase: n= 125; intervention phase: IG[5]: n=61; CG[6]: n=53	03/2011-06/2012); 16 months	Exclusion: advanced dementia, severe delirium pre-operatively; end stage		

The responsible "delirium nurse" was referred as a "liaison nurse". However, the preventive interventions were not performed as part of a liaison service. Rather, the "delirium nurse" was a member of the ward team.

Supplement No. 3

Claudia Eckstein[1], Heinrich Burkhardt[2]
1 Network Ageing Research, University of Heidelberg, Germany.
2 Department of Geriatric Medicine, University Medicine Mannheim, Germany.

No.	Study	Country	Objectives	Design	Setting[1]	Patient-sample[1]	Duration	In-/exclusion criteria	Inclusion of PwD[2]/ MCI[3] ?	Performed in geriatric ward?
13	Kurrle et al. (2019)	Australia	Evaluation of a model of care (CHOPs: Confused Hospitalised Older Persons Program) to improve recognition, assessment and management of older patients with cognitive impairment	Pre- and post-test design	6 hospitals (13 wards, 2 of those: aged care wards)	Population: all patients over 65 years and older (Aboriginal patients over 45 years); randomly selected medical records; sample: n=743	12 months (period varied between 02/2014-10/2016)	Exclusion: emergency only admissions or day only patient admission	(X)	(X)
14	Lundström et al. (1999)	Sweden	Investigation of a reorganized nursing program to reduce the incidence of post-operative delirium and to improve patient´s functional outcomes	Prospective observational study (historical comparison)	1 ortho-geriatric rehabilitation	Population: all patients (age range: 65-98, mean: 79.7 years, SD ±7.5) with femoral neck fractures; n=49	01/1993-12/1993; 12 months	Exclusion: patients without surgical treatment	(X)	X
15	Lundström et al. (2005)	Sweden	Investigation whether an education program and a reorganization of nursing/medical care would improve outcome for older delirious patients	Prospective observational study	2 general internal medicine wards	Population: age over 70 years and older; IG[5]: n=200; CG[6]: n=200	8 months (period of time not stated)	Exclusion: younger than 70 years	(X)	
16	Lundström et al. (2007)	Sweden	Examination whether a post-operative multifactorial intervention program can reduce delirium and improve patient´s outcome	RCT[4] (power-calculation conducted)	1 geriatric and 1 orthopaedic ward	Population: all patients over 70 years and older with femoral neck fractures; IG[5]: n=102; CG[6]: n=97	05/2000-12/2002; 32 months	Exclusion: severe rheumatoid arthritis, severe hip osteoar-thritis, severe renal failure, pathological fracture, bedridden before fracture	(X)	X
17	Mattison et al. (2014)	USA	Investigation whether a bundled intervention can increase detection of delirium and facilitate safer use of high-risk medications (multicomponent, pharma- and non-pharmacological intervention)	Pre- and post-test design	Medical/ surgical center geriatric-focused acute-care	Population: all patients cohort I: aged 80 and over: pre-phase: n=5.077; post-phase: n=5.571; cohort II: aged 70 to 79: pre-phase: n=4.819; post-phase: n=4.482	05/2008-09/2011; 41 months	Exclusion: other wards (ICU, oncology, psychiatry)		(X)
18	Milisen et al. (2001)	Belgium	Development and testing the effectiveness of a nurse-led interdisciplinary intervention program for delirium	Pre- and post-test design	2 trauma-tological wards	Population: patients admitted to emergency department with a traumatic fracture of prox. femur who received surgery within 24 h; IG[5]: n=60 (median age: 82 years, IQR 13); CG[6]: n=60 (median age: 80 years, IQR 12)	09/1996-03/1998; 19 months	Exclusion: multiple trauma, concussion of the brain, pathological fractures, surgery more than 72 h after admission, aphasia, blind-/deafness, and fewer than 9 years of formal education	(X)	
19	Miller et al. (2004)	USA	Testing of intervention that obtains personal information from caregivers on older adults' behaviours indicating discomfort	Pre- and post-test design	1 geriatric and 1 orthopaedic ward	Population: cohort I: age 64 and over (admitted from nursing home), cohort II: 74 years and older (admitted from a residence, foster care/ retirement community); IG[4]: n=43; CG[6]: n=38	2 months (period of time not stated)	Inclusion: diagnosis of chronic cognitive impairment/dementia, no diagnosis of cognitive impairment but mental problems noted at admission	X	X
20	Pitkälä et al. (2006)	Finland	Investigation of the effectiveness in reducing mortality of a geriatric assessment/tailored treatment for patients with delirium	RCT[4]	6 medical wards with geriatric care	Population: age over 69 years and older; IG[5]: n=87; CG[6]: n=87	09/2001 - 11/2002; 16 months	Exclusion: life expectancy less than 6 months, admission from long-term care, discharged in 48 h; inclusion: delirium	(X)	(X)
21	Robinson et al. (2008)	USA	Investigation of the effectiveness of a protocol designed to prevent older inpatients with risk factors from delirium	Pre- and post-test design	1 renal unit	Population: age over 69 years and older; pre-phase: n=80; post- phase: n=80	1 year (period of time not stated)	Inclusion: patients with any combination (dementia, vision-hearing-mobility impairment)	(X)	
22	Rudolph et al. (2014)	USA	Improvement project to identify and modify delirium risk and discharge to rehabilitation	Propensity-matched cohort study	2 acute-care medical wards	Population: veterans age over 65 years and older; IG[5]: n=566; CG[6]: n=566	Not stated	Exclusion: ICU, unable to communicate, in-patient for 48 h or longer before screening	(x)	
23	Vidán et al. (2009)	Spain	Analysing the effectiveness of a multicomponent intervention, integrated into daily practice to prevent older inpatients of delirium	Prospective cohort study	1 geriatric & 2 internal wards	Population: age over 70 years and older; IG[5]: n=140; CG[6]: n=222	01/2007-12/2007; 12 months	Exclusion: severe dementia, aphasia, coma, agonic status, expected stay shorter than 48 h	(X)	X
24	Wand et al. (2014)	Australia	Evaluation of the effectiveness of an educational program in preventing delirium in hospitalized older patients and improving staff practice	Pre- and post-test design	1 general medical ward	Population: age over 65 years and older; pre-phase: n=126; post-phase: n=129	05/2011-09/2012; 17 months	Exclusion: non-verbal patients, terminal illness, and receiving comfort care	(X)	
25	Wanich et al. (1992)	USA	Examination of targeted multicomponent interventions (strategies to educate nurses, mobilize patients, monitor medication, environmental/ sensory modifications, improve clinical/functional outcomes	Pre- and post-test design	3 medical wards	Population: age over 70 years and older; pre-phase: n=100; post- phase: n=129	06/1986-03/1987; 10 months	Exclusion: patients who were transferred from another unit or admitted for a short stay, terminal care		

Legend of above table:

(1) The exact designations of the settings and patient-samples were adopted from the original data of the studies. (The "geriatric setting" include the following terms (synonymous used): acute care for elders, acute care geriatric, acute care of elder(s) units, acute geriatric ward, care wards – specialized for elderly, elderly care, geriatric ward, ortho-geriatric rehabilitation (the latter unit does not designate a classical rehabilitation facility (exclusion criterion). This term refers to geriatric early rehabilitation in the acute hospital).
(2) PwD People with Dementia
(3) MCI Mild Cognitive Impairment
(4) RCT Randomized Controlled Trial
(5) IG Intervention Group
(6) CG Control Group
X People with dementia or cognitive impairment were exclusively included; studies performed in the geriatric setting.
(X) Studies also included participants with pre-existing dementia or cognitive impairment in their study population; studies were partly conducted in the acute geriatric setting; studies were performed in non-geriatric units/regular wards with a geriatric care concept.
