# Supplementary material for: Multicomponent, nonpharmacological delirium interventions for older inpatients: A scoping review
Source: Z Gerontol Geriatr. 2019 Oct 18;52(Suppl 4):229–42. doi: 10.1007/s00391-019-01627-y (PMC6820613; doi:10.1007/s00391-019-01627-y)
Supplement: Supplementary file 4 — 4. Identified multicomponent, nonpharmacological interventions and patient outcomes [file 391_2019_1627_MOESM4_ESM.pdf]

ZGG_Zeitschrift für Gerontologie und Geriatrie
Multicomponent, non-pharmacological delirium interventions for older inpatients. A scoping review.

Supplement No. 4

Claudia Eckstein[1], Heinrich Burkhardt[2]
1 Network Ageing Research, University of Heidelberg, Germany.
2 Department of Geriatric Medicine, University Medicine Mannheim, Germany.

Identified multicomponent, non-pharmacological interventions and patient outcomes

No.	Study	**Type of program** P = Prevention M = Management	Core components of the delirium intervention programs																		Additional interventions	Main patient outcomes
			Detection	Mode of health care supply	Physical environment	Social environment	Orientation	Cognitive stimulation	Sensory impairments	Fluid balance	Nutrition/eating/metabolism	Infection	Mobilization	Medication review	(Emergency) Surgery	Pain	Day-night rhythm	Monitoring	Staff-education	Sufficient oxygen supply		
1	Allen et al. (2011)	P & M	Six-Item Screener, NU-DESC	Multidisciplinary/ interprofessional				X	X	X			X	X			X	X	X		Computerized treatment order set: with standardized diagnostic, procedures	• Incidence of delirium: IG(1): 7.2% vs. CG(2): 8.8% (1.6% difference; 95% CI=–5.9% to 9.1%)<br>• Length of stay: IG(1) 4.0 vs. CG(2): 7.6 days (3.6-day difference; 95% CI= 0.66 to 6.49)<br>• Post-implementation outcomes: fewer deaths: 23% vs. 9.5%; transfers to ICU: 18% vs. 0%; fewer 30-day readmissions; 31% to 5%; higher percentage of post-implementation patients had antipsychotic medication administered during stay
2	Andro et al. (2012)	P	No routine screening; study-phase: CAM	Multidisciplinary/ interprofessional	X	X	X		X	X	X		X	X		X						• Incidence of delirium: IG(1) : 5.26% vs. CG(2): 15.45%, (RR 0.34 CI 95% 0.15-0.78)<br>• MMSE: Pre-phase: mean MMSE 14.3 ± 7.8; post-phase: mean MMSE 13.9 ± 6.9
3	Avendaño Céspedes et al. (2016)	P & M	CAM	Nurse-led intervention	X	X	X		X	X	X	X	X	X		X	X	X	X	X	Caregiver booklet	• Prevalence of delirium: IG(1): 33.3% vs. CG(2): 48.3% (RR 0.54; 95% CI 0.17–1.72)<br>• Incidence of delirium: IG(1): 14.3% vs. CG(2): 41.4% (RR 0.24; 95% CI 0.06–0.99; p=.039)<br>• Duration of delirium: IG(1): 1.7 (range1-6) vs. CG(2): 3.4 days (range 1-13) (p=.063)<br>• Severity of delirium: IG(1): 35.0 vs. CG(2): 65.0 (mean difference 30.0, 95% CI 1.5-58.5, p=.040), but mean severity per day was higher in the IG(1) (21.1 vs. 18.6)<br>• Mortality: delirious patients compared to patients without delirium at admission: 33.3% vs. 14.6%, but without differences in: IG(1) and CG(2)<br>• Length of stay: patients with delirium compared to those without delirium: 7.7 (SD 4.1) vs. 7.1 (SD 4.2) days
4	Benedict et al. (2009)	P	NEECHAM (modified)	Multidisciplinary/ interprofessional	X	X	X		X	X	X		X	X			X	X	X		Caregiver booklet, geriatric screening: GDS, alcohol-anamnesis	• Incidence of delirium: no effect<br>• Mean of modified NEECHAM on day 3 for the IG(1) was 3.76 vs. CG(2): 3.24, p=.368;<br>• Medication: anticholinergic during hospital stay: IG(1): 14% vs. CG(2): 9%<br>• Length of stay: IG (1) 6.0 vs. CG(2): 5.8<br>• Functional deficits: IG(1): 77% vs. CG(2): 61%
5	Bo et al. (2009)	P	No routine screening; study-phase: CAM/DRS	Multidisciplinary/ interprofessional	X		X			X	X		X	X		X	X					• Incidence of delirium: IG(1): 6.6% vs. CG(2): 15.2% (adjusted: RR: 0.90, 95% CI 0.024-0.331, p<.001 (setting was independently associated with lower incidence, p <.001)<br>• Length of stay: all delirious patients stayed longer (12.3 -f 3.4 vs. 6.3 + 2.0 d, p <0.001).
6	Foster et al. (2010)	P & M	Abbreviated mental test; study-phase: CAM	Multidisciplinary/ interprofessional	X	X	X	X						X					X		Clinical pathway/ standardized nursing care, “information and “rummage box”, carer leaflet	• Prevalence of delirium: similar in both groups, 34 randomly selected sets of patients’ notes were audited over a 4-week period. Nurses noted confusion in 14, physicians in 10 cases. Only 4 patients had an Abbreviated Mental Test.<br>• All patients had between 1-6 prevention strategies implemented according to the care plan
7	Godfrey et al. (2013)	P		Multidisciplinary/ interprofessional	X	X	X	X	X	X	X	X	X	X		X	X		X	X		*Not focused on patient-outcomes*
8	Hasemann et al. (2016)	P & M	DOS; clock test, MSQ; study-phase: CAM, DRS-R-98	(Nurse-led) Multidisciplinary/ interprofessional	X	X	X	X	X	X	X	X	X	X	X	X	X	X	X	X	Advanced screening: digit span/ clock-/ comprehension test, MMSE, individual intervention plan	• Incidence of delirium: 3,6% lower in IG(1), but not significantly (p=.896)<br>• Severity of delirium: no significant effect IG(1): vs. CG(2) (F(1,102) = 0.093; p=.761)<br>• Duration of delirium: IG(1): 4.1 days (SD 3.4) vs. CG(2): 3.0 days (SD 3.1)<br>• Adherence vs. non-adherence: (F(1.48) = 4.079, p=.050 (overall non-adherence rate: 34.1%)<br>• Shift from neuroleptic to atypical neuroleptic less Lorazepam use (U=361.5; p=.027)
9	Holroyd-Leduc et al. (2010)	P	CAM	Multidisciplinary/ interprofessional					X	X	X			X	X	X		X	X		Study	• Incidence of delirium: no effect on the over-all delirium rate (33% pre vs. 31% post, p=.84), differences in enrolled hospitals<br>• Mortality: no pre-post differences (one death among those enrolled)<br>• Length of stay: no significant differences in 12 days post vs. 14 days pre; p=.74<br>• Falls: 6% post vs.10% pre; p=.43<br>• Discharges to long-term care: 6% post vs. 13% pre; p=.20
10	Holt et al. (2013)	P	No routine screening; study-phase: CAM/ DRS-R-98	Multidisciplinary/ interprofessional			X		X	X	X		X			X			X			• Delirium incidence during first 7 days: IG(1): 4.6% vs. CG(2): 13.3%, p=.006<br>• Duration of delirium: IG(1): 0.06 days, ± 0.287 vs. CG(2): 0.29 days; ± 0.931, p=.002<br>• Severity of delirium: IG(1): 9.17, ± 7.94 vs. CG(2): 16.86, ± 4.92; p=.005<br>• Mortality/length of hospital stay/functional status at discharge/admission to long-term care: no significant group differences<br>• Readmission: CG(2) were significantly higher

Claudia Eckstein[1], Heinrich Burkhardt[2]
1 Network Ageing Research, University of Heidelberg, Germany.
2 Department of Geriatric Medicine, University Medicine Mannheim, Germany.

No.	Study	**Type of program** P = Prevention M = Management	Core components of the delirium intervention programs																		Additional interventions	Main patient outcomes
			Detection	Mode of health care supply	Physical environment	Social environment	Orientation	Cognitive stimulation	Sensory impairments	Fluid balance	Nutrition/eating/metabolism	Infection	Mobilization	Medication review	(Emergency) Surgery	Pain	Day-night rhythm	Monitoring	Education	Sufficient oxygen supply		
11	Jeffs et al. (2013)	P	No routine screening; study-phase: CAM/ MMSE, clock test	Multidisciplinary/ interprofessional		X	X						X									• Incidence of delirium: IG$^{(1)}$: 4.9%, 95 % CI 2.3%-7.3% vs. CG$^{(2)}$: 5.9% 95% CI 3.8- 9.2%<br>• Duration of delirium: IG$^{(1)}$: median: 2.4 (IQR: 0.9-8.9) vs. CG$^{(2)}$: 2.1 (IQR: 1.4–6.6) days, p=0.9<br>• Severity of delirium: IG$^{(1)}$: median 3.0 (IQR: 3.0-5.0) vs. CG$^{(2)}$: 4.0 (IQR: 3.0-4.5), p=0.7<br>• Discharge destination/length of stay: no effect
12	Kratz et al. (2015)	P	DOS; study-phase: other several tests	Multidisciplinary/ interprofessional		X	X	X	X	X	X		X				X		X		Individual intervention plan, validation method	• Incidence of post-operative delirium: IG$^{(1)}$: 4.9% (95% CI 0.0-11.5) vs. CG$^{(2)}$: 20.8% (95% CI 11.3-32.1), p=.046 (pre-intervention: 20.2%)<br>• Important predictors of post-operative delirium: low score of MMSE, advanced age, preoperative infection
13	Kurrle et al. (2019)	P & M	Cognitive screening: AMTS and further tests; delirium screening:, DRAT, CAM	Multidisciplinary/ interprofessional	X	X	X	X	X	X	X	X	X	X	X	X	X	X	X	X	Avoiding physical restrains, CHOPs icons/magnets/ posters, advanced risk assessment, family information, education and involvement, considering anxiety, fall prevention	• Cognitive screening at admission: IG$^{(1)}$: 37 vs. CG$^{(2)}$: 29 (OR = 1.42, 1.04-2.72)<br>• Cognitive screening within 24 h: IG$^{(1)}$: 31 vs. CG$^{(2)}$: 12 (OR = 3.32, 2.50-4.90)<br>• DRAT completed: IG$^{(1)}$: 43 vs. CG$^{(2)}$: 31 (OR = 1.73, 1.28-2.33)<br>• Delirium risk identified: IG$^{(1)}$: 48 vs. CG$^{(2)}$: 19 (OR = 4.04, 2.89-5.64)<br>• Cognitive screening repeated: IG$^{(1)}$: 28 vs. CG$^{(2)}$: 15 (OR = 2.25, 1.56-3.25)<br>• Delirium assessment conducted: IG$^{(1)}$: 56 vs. CG$^{(2)}$: 33 (OR = 2.55, 1.90-3.43)<br>• Antipsychotics prescribed: IG$^{(1)}$: 21 vs. CG$^{(2)}$: 26 (OR = 1.34, 0.71-1.88)<br>• Physical restraints used: IG$^{(1)}$: 4 vs. CG$^{(2)}$: 8 (OR = 0.54, 0.29-0.99)<br>• Interaction with families: IG$^{(1)}$: 64 vs. CG$^{(2)}$: 39 (OR = 2.81, 2.09-3.79)<br>• Delirium coded at discharge: IG$^{(1)}$: 79 vs. CG$^{(2)}$: 27 (OR = 10.2, 7.23-14.2)
14	Lundström et al. (1999)	P & M	No routine screening; study-phase: OBS-scale	Multidisciplinary/ interprofessional	X	X					X		X		X	X		X	X	X	Individual intervention plan, team collectively formulates goals with patients, 2 contact persons	*Comparison to previous studies I and II:*<br>• Prevalence of delirium: IG$^{(1)}$: 20.4% vs. CGI$^{(2)}$: 33.3% (p.098), CGI$^{(2)}$: 29.1% (p=.253);<br>• Post-operative incidence: IG$^{(1)}$: 30.6% vs. CGI$^{(2)}$: 61.3% (p<.001), CGII^{2}: 47.6% (p=.047); stay: IG$^{(1)}$: 12.5 vs. CGI$^{(2)}$: 17.4, CGII$^{(2)}$: 11.6 days<br>• Duration of delirium: IG$^{(1)}$: 16.3% vs. CGI$^{(2)}$: 39.6% (p=.004), CGII$^{(2)}$: 29.1% (p=.088)<br>• In-hospital mortality: IG$^{(1)}$: 2.0% vs. CGI$^{(2)}$: 2.7% (p=.805), CGII$^{(2)}$: 5.8%, (p=.298)<br>• Six-month mortality: IG$^{(1)}$: 16.3% vs. CGI$^{(2)}$: 16.2% (p=.986), CGII$^{(2)}$: 12.6%, (p=.536)<br>*Comparison to previous study III:*<br>• Incidence of delirium: lower post-operative delirium (26.7% vs. 42.9%, p.129)<br>• Better walking abilities and living conditions<br>*Comparison to previous studies IV and V:*<br>• Incidence of delirium: IG$^{(1)}$: 21.0% vs. CGIV$^{(2)}$: 52.0% (p<.001), CGV$^{(2)}$: 44.0% (p=.014)
15	Lundström et al. (2005)	P & M	No routine screening; study-phase: OBS-scale, MMSE	Multidisciplinary/ interprofessional		X					X	X	X	X					X	X	Reorganisation patient allocation system with individual care, treat/assess underlying causes	• Prevalence of delirium: equally on admission and on day 7 in IG$^{(1)}$ (n=19/63, 30.2% vs. CG$^{(2)}$: n=37/ 62, 59.7%, p=.001)<br>• Duration of delirium: IG$^{(1)}$: 10.8, ± 8.3 vs. CG$^{(2)}$: 20.5, ± 17.2 days, p<.001<br>• No patient with dementia remained delirious on day 7 in IG$^{(1)}$ compared with four patients still delirious on day 7 in CG$^{(2)}$<br>• Length of stay: IG$^{(1)}$: 9.4 ± 8.2 vs. CG$^{(2)}$: 13.4 ± 12.3 days, p=.001); delirious patients: IG$^{(1)}$: 10.8 ± 8.3 vs. CG$^{(2)}$: 20.5 ± 17.2 days, p<.001<br>• Mortality: 2 delirious patients in IG$^{(1)}$ and 9 in CG$^{(2)}$ died during hospitalization (p=.03)
16	Lundström et al. (2007)	P & M	No routine screening; study-phase: OBS-scale, MMSE, GDS-15	Multidisciplinary/ interprofessional							X	X	X	X	X	X	X	X	X	X	Individual intervention plan, teamwork explicitly named as intervention, prevention of decubiti, osteoporosis, falls, rehabilitation, advanced mobilization	• Incidence of delirium: IG$^{(1)}$: 54.9% vs. CG$^{(2)}$: 75.3%, p=.003; IG$^{(1)}$: 18% vs. CG$^{(2)}$: 52 % were delirious after 7. Post-operative day, p<.007<br>• Prevalence of delirium: IG$^{(1)}$: 21.8% vs. CG$^{(2)}$: 30.9%, p=.144<br>• Duration of delirium: IG$^{(1)}$: 5.0, ± 7.1 vs. CG$^{(2)}$: 10.2, ± 13.3 days, p=.009<br>• Duration of delirium: post-operative delirium in patients with dementia was in IG$^{(1)}$: 3.2 ± 4.1 vs. CG$^{(2)}$ 12.8 ± 17.6 days in CG$^{(2)}$ (p=.003) (15 patients with dementia in CG$^{(2)}$ were delirious on discharge, 9 from IG$^{(1)}$ (p<.001)<br>• Mortality: did not differ during hospitalization and at the 4-and 12-month follow-ups between intervention and control samples<br>• Patients from IG$^{(1)}$ had fewer complications, such as decubitus ulcers, urinary tract infections, nutritional complications, sleeping problems and falls than CG$^{(2)}$<br>• Length of stay: hospitalization was shorter in IG$^{(1)}$ than in the CG$^{(2)}$ (28.0 ± 17.9 days vs. 38.0 ± 40.6 days, p=.028
17	Mattison et al. (2014)	P & M	RASS, CAM	Multidisciplinary/ interprofessional	X								X	X			X	X	X		Computerized physician-order entry system, checklist to accompany standard bedside monitoring, daily review	• In-hospital mortality: Age class 70-79: IG$^{(1)}$: 1.7% vs. CG$^{(2)}$: 1.7%; age class ≥ 80: IG$^{(1)}$: 2.5% vs. CG$^{(2)}$: 2.8%; p=.39<br>• The number of orders to activate the rapid response team increased in participants receiving the bundle and in controls (differences OR=1.23, 95% CI 0.68-2.24, p=.49)<br>• Participants receiving the bundle had less haloperidol (OR=0.60, 95% CI 0.39-0.91, p=.02) and less morphine (OR=0.52, 95% CI 0.42-0.63, p<.001)<br>• Participants who received bundle were more likely to be discharged home (OR=1.18, 95% CI1.04-1.35, p=.01)

Claudia Eckstein[1], Heinrich Burkhardt[2]
1 Network Ageing Research, University of Heidelberg, Germany.
2 Department of Geriatric Medicine, University Medicine Mannheim, Germany.

No.	Study	**Type of program** P = Prevention M = Management	Core components of the delirium intervention programs																		Additional interventions	Main patient outcomes
			Detection	Mode of health care supply	Physical environment	Social environment	Orientation	Cognitive stimulation	Sensory impairments	Fluid balance	Nutrition/eating/metabolism	Infection	Mobilization	Medication review	(Emergency) Surgery	Pain	Day-night rhythm	Monitoring	Education	Sufficient oxygen supply		
18	Milisen et al. (2001)	P & M	NEECHAM; study-phase: CAM	Nurse-led interdisciplinary intervention program										X				X	X		Standard nursing care plan, “resource nurse for delirium”	• Incidence of delirium: IG(1): 20.0% vs. CG(2): 23.3%, p.82<br>• Duration of delirium: IG(1): median: 1 day vs. CG(2): median: 4 days (IQR=5.5), p=.03)<br>• Severity of delirium: less (p=.0049) in IG(1)<br>• Length of stay: trend toward decreased stay<br>• Cognitive functioning: only a difference in the sub dimension “memory” in the IG(1) (p=.0357)<br>• Functional status: no effects<br>• Mortality: results were inconclusive
19	Miller et al. (2004)	P & M	No routine screening; study-phase: NEECHAM, Katz Index, discomfort assessment with DS-DAT	Nurse-led intervention	X	X			X	X	X		X			X	X	X	X		Elder care supportive protocol (client profile, individualized care, elder guide), observation checklist, advanced caregiver involvement	• Patient´s discomfort: The IG(1) had significantly less discomfort at T2 (mean, 6.38) than the baseline group (mean, 8.25) (independent samples t-test, r[50] = 2.70, p=.047; but after repeated measures ANOVA, these differences were no longer significant (p=.075)<br>• Further outcome variable was described: family involvement<br>• no significant differences between baseline and treatment conditions for the outcome variables of physical function, acute confusion, and length of stay
20	Pitkälä et al. (2006)	M	Routine: MMSE; study-phase: CAM, MMSE, digit span, proxy interview	Multidisciplinary/ interprofessional				X			X		X	X					X		Individually tailored comprehensive geriatric assessment/ treatment, detailed diagnostics of underlying causes, discharge planning	• Cognitive status: cognition, according to MMSE score had significantly improved after baseline (IG(1):18.4 vs. CG(2): 15.8, p=.047)<br>• Complications during hospitalization (e.g. new fractures): IG(1): 16.1 vs. CG(2): 19.1<br>• Mortality (1-year follow-up): IG(1) : 60.0% vs. CG(2): 64.4%, p=.638<br>• Permanent institutional care (1-year follow-up): IG(1) : 42.5% vs. CG(2): 51.7%, p=.224<br>• Medication: IG(1) received significantly more acetylcholinesterase inhibitors (58.6% vs. 9.2%), atypical antipsychotics (69.8% vs. 30.2%), and fewer conventional neuroleptics (8.0% vs. 23.0%) than CG(2)
21	Robinson et al. (2008)	P	Retrospective chart-based method (68)	Nurse-led intervention	X	X			X				X			X			X		Caregiver information (ADL´s); portable amplifying device	• Incidence of delirium: IG(1): 13.8% vs. CG(2): 37.5%, p>.001<br>• Subgroup analyses people with dementia: 12 subjects (with various other risk factors) six of the pre-intervention group developed delirium, only 1 in the post-intervention group
22	Rudolph et al. (2014)	P	Days of the week/ months of the year backward, clock test	Nurse-led intervention				X	X								X		X		Education for patients and family members	• Length of stay: IG(1): 4.4 days vs. CG(2): 5.1 days<br>• Restraint use: IG(1): 2.8% vs. CG(2): 6.9%<br>• Rehabilitation discharge: IG(1): 20.1% vs. CG(2): 17.9%<br>• Cost: IG(1): 9.446 $ vs. CG(2): 10.836 $
23	Vidán et al. (2009)	P	No routine screening; study-phase: CAM	Multidisciplinary/ interprofessional			X		X	X	X		X	X			X		X		Caregiver letter, advanced geriatric assessment	• Incidence of delirium: IG(1): 11.7% vs. CG(2): 18.5%, p=.04, RR 37 % (after adjustment: intervention was associated with lower incidence: OR=0.4, 95 %, CI 0.24-0.77, p=.005<br>• Duration of delirium: IG(1): 31.1 ± 43.0 vs. CG(2): 33.6 ± 22.0, p=.73<br>• Severity of delirium: IG(1): 4.9 ± 0.4 vs. CG(2): 5.3 ± 1.0, p=.08<br>• Length of delirium episode, hours: IG(1): 31.1 ± 43.0 vs. CG(2): 33.6 ± 22.0, p=.73<br>• Recurrence of delirium: IG(1): 0/20 vs. CG(2): 6/69, p=.22<br>• Functional decline in patients with delirium: IG(1): 9(60.0) vs. CG(2): 37(71.2), p=.041<br>• Mortality: IG(1): n=2/20 vs. CG:(2) n=10/69, p=.60
24	Wand et al. (2014)	P & M	No routine screening; study-phase: CAM, MMSE, RUDAS, Barthel	Multi-disciplinary/ interprofessional	X				X	X	X	X	X	X		X	X		X	X	Avoidance of physical restraints	• Incidence of delirium: IG(1) : 10.1% vs. CG(2): 19.0%, X2=4.14, p=.042<br>• Mortality: IG(1) : 4 vs. CG(2): 1 inpatient death, no differences in death between the groups<br>• Functional status (on discharge): improved in IG(1) (mean improvement 5.3 points, p<.001, SD 13.31, 95% CI -7.61 to -2.97, not seen in the CG(2)
25	Wanich et al. (1992)	P & M	No routine screening; study-phase: MMSE, BPRS, Katz Index	Nurse-led intervention	X	X	X						X	X					X		Caregiver education, discharge planning	• Incidence of delirium: IG(1): 19% vs. CG(2): 22%, p=.61<br>• Functional status: (1) better: IG(1): 21% vs. CG(2): 10%; same: IG(1): 69% vs. CG(2): 74%;<br>• Worse: IG(1): 13% vs. CG(2): 16%<br>• Complication during hospitalization: IG(1): 19% vs. CG(2): 16%, p=.62<br>• Mortality: IG(1): 8% vs. CG(2): 5%, p=.36<br>• Length of stay/admission to long-term care/ number of complications: no significant difference in both groups

Legend of above table:

(1) IG Intervention Group
(2) CG Controll Group;
X Intervention component integrated or as a risk factor considered
