# Supplementary material for: Multicomponent, nonpharmacological delirium interventions for older inpatients: A scoping review
Source: Z Gerontol Geriatr. 2019 Oct 18;52(Suppl 4):229–42. doi: 10.1007/s00391-019-01627-y (PMC6820613; doi:10.1007/s00391-019-01627-y)
Supplement: Supplementary file 5 — 5. Recommendations (overview) of relevant medical delirium guidelines [file 391_2019_1627_MOESM5_ESM.pdf]

ZGG_Zeitschrift für Gerontologie und Geriatrie
Multicomponent, non-pharmacological delirium interventions for older inpatients. A scoping review.

Supplement
No. 5

Claudia Eckstein[1], Heinrich Burkhardt[2]
1 Network Ageing Research, University of Heidelberg, Germany.
2 Department of Geriatric Medicine, University Medicine Mannheim, Germany.

Recommendations (overview) of relevant medical delirium guidelines

**Recommendations to prevent / to manage delirium**	**Number of included guidelines (GL)***									
	**1**	**2**	**3**	**4**	**5**	**6**	**7**	**8**	**9**	**10**
**1 Detection**	X	X	X	X	X	X	X	X	X	X
**2 Mode of health care supply**	X[1]	X	X		X	X	X	X	X	
**3 Material environment**		X	X	X	X	X	X	X		X
**4 Social environment**		X	X	X	X	X	X	X		
**5 Orientation**	X	X	X	X	X	X	X	X	X	X
**6 Cognitive stimulation**			X		X	X	X	X		
**7 Sensory impairments**	X	X	X	X	X	X	X	X	X	X
**8 Fluid balance**	X	X	X	X	X	X	X	X	X	X
**9 Nutrition/eating/metabolism**		X	X	X	X	X	X	X	X	
**10 Infection**	X	X	X	X	X	X	X	X	X	X
**11 Mobilization**	X	X	X	X	X	X	X	X		
**12 Medication review**	X	X	X	X	X	X	X	X	X	X
**13 (Emergency) Surgery**	X	X	X	X	X			X	X	X
**14 Pain**		X	X	X	X	X	X	X	X	X
**15 Day-night rhythm**	X	X	X	X		X	X	X		
**16 Monitoring**	X	X	X	X	X	X	X	X	X	X
**17 Education**		X	X	X		X	X	X	X	X
**18 Sufficient oxygen supply**		X	X	X	X	X	X	X	X	X

Legend of this and all following tables: X = Intervention component integrated or as a risk factor considered

***Included medical delirium guidelines (GL) in this and all following tables:**

1	International:	Leentjens et al. (2012)
2	Australia:	Health Care of Older Australian Standing Committee (HCOASC), and the Astralian Department of Health and Ageing (2008)
3	Canada:	Canadian Coalition for Seniors' Mental Health (CCSMH) (2006)
4	Great Britain:	British Geriatrics Society (BGS), Royal College of Physicians (RCP) (2006)
5	Great Britain:	Fleet, J.; Ernst,T. Guy´s and St. Thomas´- NHS Foundation Trust (2013)
6	Great Britain:	NICE (National Institute for Health and Care Excellence, GB (2010)
7	Netherlands:	Leentjens et al. (2014)
8	Switzerland:	Savaskan et al. 2016)
9	USA:	The American Geriatrics Society Expert Panel on Postoperative Delirium in Older Adults (2015)
10	USA:	American Psychiatric Association (APA) (2010)

[1] Available evidence does not allow recommendations of specific models of organization (GL: 1, Leentjens et al. 2012)

ZGG_Zeitschrift für Gerontologie und Geriatrie
Multicomponent, non-pharmacological delirium interventions for older inpatients. A scoping review.

Claudia Eckstein[1], Heinrich Burkhardt[2]
1 Network Ageing Research, University of Heidelberg, Germany.
2 Department of Geriatric Medicine, University Medicine Mannheim, Germany.

Recommendations (details) of relevant medical delirium guidelines

**Recommendations to prevent/to manage delirium**	**Number of included guideline (GL)***									
	**1**	**2**	**3**	**4**	**5**	**6**	**7**	**8**	**9**	**10**
**1 Detection**										
Defined as risk groups (old age, people with dementia/MCI)	X	X	X	X	X	X	X	X	X	X
Screening/early detection	X	X	X	X	X	X	X	X	X	X
Pay attention to changes in behavior								X		
Targeting individual risk factors			X							
Medical report								X		
History from relatives/carers/examination/ investigation				X						
Professionals are familiar to persons at risk					X	X	X			
Follow up and assess for possible dementia						X	X		X	
**2 Mode of health care supply**										
No changes of nurses, wards, bays					X	X	X			
Carry out multicomponent interventions…		X	X			X	X	X	X	
…that are tailored, based on assessments						X	X			
…that are on a multidisciplinary/ interprofessional basis by (competent and trained health care professionals)			X			X	X	X	X	
Don´t do unnecessary tests					X					
Prevent complications		X								
Available evidence does not allow for recommen-dations of specific model of organization	X									
**3 Material environment**										
Personal objectives/family pictures		X			X					
Avoid bedrails						X	X			
Avoid physical restraints		X		X	X			X		
Avoid sensory deprivation			X							X
Quiet (especially at night)/safe environment		X			X	X	X	X		X
Prefer single rooms		X								
Avoid side rooms					X					
Put delirious patients not in the same room			X							
Optimal level of environmental stimulation										X
Environmental risk factors should be modified/ incorporated into care plans		X	X							
Reduce/eliminate environmental factors			X							X
Appropriate Lighting		X	X		X	X	X	X		
…and avoid bright lights at night					X			X		
**4 Social environment**										
Empathy/notice (acknowledge feelings,…)					X			X		
Attitude of warmth, calmness and kind firmness, explain providing care			X							
Customized communication			X	X	X	X	X	X		
Discuss topics that are familiar or of interest			X							
Encourage independence (in basic ADLs)		X								
Avoid gestures or touching			X							
Family/carer involvement		X			X	X	X	X		
Alliance with patients/carer/friends			X							
Allow watching movies, listening to music – interrupt if patient is disorientated or disturbed			X							

Claudia Eckstein[1], Heinrich Burkhardt[2]
1 Network Ageing Research, University of Heidelberg, Germany.
2 Department of Geriatric Medicine, University Medicine Mannheim, Germany.

**Recommendations to prevent / to manage delirium**	**Number of included guideline (GL)***									
	**1**	**2**	**3**	**4**	**5**	**6**	**7**	**8**	**9**	**10**
**5 Orientation**										
(Re-)Orientation (information staff/role/who and where they are,…	X	X	X	X	X	X	X	X	X	X
Orientation protocol			X							
Help with repeated questions								X		
Clock, calendar		X			X	X	X	X		X
Clear signage						X	X	X		X
Engagements in activities				X						
**6 Cognitive stimulation**										
General recommendations – not specified			X		X	X	X	X	X	
Avoid over-stimulation								X		
**7 Sensory impairments**										
Ensure/assist wearing vision/hearing aids	X	X	X	X	X	X	X	X	X	X
Evaluating reversible causes/devices			X			X	X			
**8 Fluid balance**										
Reducing the risk/treatment of dehydration	X	X	X	X	X	X	X	X	X	X
Ensure/encourage/assist oral drinking		X	X		X	X	X	X		
s.c./i.v. fluid if necessary						X	X	X		
Early remove of i.v. infusion								X		
**9 Nutrition/Eating/Metabolism**										
Balanced diet/avoid/treat malnutrition		X	X	X	X	X	X	X	X	
Documentation of food intake								X		
Involving diatetics if necessary					X			X		
Nutrition support						X	X			
Encourage patients to move to dining room								X		
Invite relatives to share mealtime								X		
Consider nutrition by providing dentures					X	X	X	X		
Avoiding soor/aspiration pneumonia								X		
Balancing Electrolyte		X	X	X	X	X	X	X		X
Regulation of bowel function/prevention of										
constipation		X			X	X	X	X	X	
Regulation of bladder function		X	X	X		X	X	X		
**10 Infection**										
Looking for and treating infection	X	X	X	X	X	X	X	X	X	X
Avoiding iatrogenic events				X	X			X		
Avoiding (indwelling) catheter		X	X	X	X	X	X	X		
**11 Mobilization**										
Encourage/assist regular mobilization		X		X	X			X		
Range of motion exercise						X	X			
Exercise in bed								X		
Supported by physio-/occupational therapist								X		
Fall prevention/improving sense of balance					X			X		
Early mobilization (general)…			X	X	X					
…soon after surgery						X	X	X		
Provide appropriate walking aids						X	X	X		
Reduce the risk of immobility	X	X		X						

ZGG_Zeitschrift für Gerontologie und Geriatrie
Multicomponent, non-pharmacological delirium interventions for older inpatients. A scoping review.

Claudia Eckstein[1], Heinrich Burkhardt[2]
1 Network Ageing Research, University of Heidelberg, Germany.
2 Department of Geriatric Medicine, University Medicine Mannheim, Germany.

**Recommendations to prevent / to manage delirium**	**Number of included guideline (GL)***									
	**1**	**2**	**3**	**4**	**5**	**6**	**7**	**8**	**9**	**10**
**12 Medication review**										
Reduce/avoid polypharmacy			X	X		X	X			
Medication control		X	X			X	X	X	X	X
…administer only essential medications								X		
Reduce/avoid /be careful with psychoactive drugs	X	X	X	X		X	X	X		
Reduce/avoid drugs that induce delirium postoperatively									X	
Monitor drug intake…								X		
…and adverse drug disease			X							
**13 (Emergency) Surgery**										
Prevent/detect/treat post-op complications			X	X		X	X	X	X	
Proactive consultation (geriatrician/psychia-trist/…) to persons undergoing surgery			X							
…preoperatively or within 24h after surgery, and daily after surgery		X								
**14 Pain**										
Assessing/treating for pain (non-verbal signs, people with dementia and communication difficulties)		X	X	X	X	X	X	X	X	X
Reviewing appropriate pain management			X			X	X	X		
Non-pharmacological approaches			X							
**15 Day-night rhythm**										
Promoting relaxing/sleep (patterns) and encouraging wakefulness	X	X	X		X	X	X	X	X	
Avoiding nursing or medical procedures during night						X	X	X		
Avoid routine use of sedatives for sleep problems – use non-pharmacologic sleep-protocol			X							
**16 Monitoring**										
(Daily), close observation of all people in hospital for recent changes or fluctuations in usual behavior	X	X	X	X	X	X	X	X	X	X
Documentation		X	X	X	X	X	X	X	X	X
Assessment (Diagnose)	X	X	X	X	X	X	X	X	X	X
**17 Education**										
Educational interventions directed to staff		X	X	X	X	X	X	X	X	
Formal programs with ongoing formal/informal refresher sessions									X	
Training to recognize hypoactive delirium									X	
Educate patients and their carers/family		X								X
**18 Sufficient oxygen supply**										
Assessing hypoxia and optimizing saturation		X	X	X	X	X	X	X	X	X

Claudia Eckstein[1], Heinrich Burkhardt[2]
1 Network Ageing Research, University of Heidelberg, Germany.
2 Department of Geriatric Medicine, University Medicine Mannheim, Germany.

**Additional recommendations for treatment of delirium**	**Number of included guideline (GL)***									
	**1**	**2**	**3**	**4**	**5**	**6**	**7**	**8**	**9**	**10**
**Assessing the psychiatric status and monitoring/psychiatric management**										x
**Increase supervision during delirium (in the following sequence: RN´s, family members, nurse sitter, personal care attendant or patient companion)**			x							
**Cognitive-emotional supportive measures**										x
**Intraoperative measurement**									x	
**Triggers and causes**										
Investigate and address causes of delirium	x	x	x	x	x	x	x	x	x	x
Treatment of underlying/contributing causes…	x		x	x	x	x	x	x		x
… or combination of causes						x	x			
Re-evaluate underlying causes						x	x			
**Signs and symptoms**										
Treatment of hypoactive delirium								x		
Coordinating the care with other clinicians, advice to nurses/general medical physicians										x
Initiating immediate (somatic) interventions for urgent general medical conditions										x
Psychiatric review, if hallucinations present					x					
Symptomatic treatment/management of symptoms	x	x								
**Advice and information**										
Explanation of the cause/the diagnoses of confusion to relatives…				x	x	x	x	x		
…and patients						x	x	x		x
Reassurance/education regarding risk factors for future episodes										x
Integration of caregivers/ family into therapy								x		
Transfer to another practitioner/ service: inform about the presence of delirium and treatment			x							
**Assessing individual and family psycho-logical/social characteristics**										x
**Establishing/ maintaining a supportive therapeutic stance with patients/family, other clinicians**										x
**Behavioral management strategies/ effective communication techniques especially to agitated patients**	x		x			x	x	x		
**De-escalation: use of verbal and non-verbal techniques if a patient is distressed/considered a risk to themselves or others**					x	x	x			
**In case that the initial management described above has no effect, some guidelines recommend a second line management for those in distress or considered to be at risk to themselves or others. If de-escalation techniques have no effect, pharmacological interventions may be considered**	x					x	x			
